# Functional analysis of the methanobactin-encoding gene cluster of *Methylocystis* sp. strain SB2

**DOI:** 10.1128/spectrum.01622-26

**Published:** 2026-09-18

**Authors:** Peng Peng, Thomas Bobik, Alan A. DiSpirito, Jeremy D. Semrau

**Affiliations:** 1 Department of Civil and Environmental Engineering, University of Michigan 542596, Ann Arbor, Michigan, USA; 2 Roy J. Carver Department of Biochemistry, Biophysics and Molecular Biology, Iowa State University 117233 , Ames, Iowa, USA; Connecticut Agricultural Experiment Station, New Haven, Connecticut, USA

**Keywords:** methanotrophs, metallophore, methanobactin, copper, Wilson disease

## Abstract

Methanobactins (MBs) are ribosomally synthesized, post-translationally modified polypeptides that have high binding affinities for copper. The key step for post-translational modification of the MB polypeptide precursor is the formation of two heterocyclic rings (pyrazinedione, imidazolone, and/or oxazolone) that are crucial for copper binding. Knowledge on genes responsible for ring formation is limited, particularly for MB from *Methylocystis* sp. strain SB2 (MB-SB2), due to a lack of genetic tools for this methanotroph. Here, we performed systematic functional analyses of the gene cluster of MB-SB2 via heterologous expression of these genes in *Methylosinus trichosporium* OB3b. We first deleted the native MB gene cluster of *M. trichosporium* OB3b and inserted the complete MB-SB2 gene cluster in its place, enabling *M. trichosporium* OB3b to produce MB-SB2. We then constructed a series of mutants, with individual genes of the MB-SB2 gene cluster deleted. Deletion of either *mbnB* or *mbnC* abolished the formation of both rings in MB-SB2. Strikingly, deletion of *mbnF* eliminated only the imidazolone ring of MB-SB2 while leaving the oxazolone ring intact. Back-complementation of *mbnB*/*C*/*F* in the respective deletion mutant restored MB-SB2 production. Deletion of *mbnM*, *mbnS*, *mbnP*, or *mbnH* did not affect ring formation but led to the formation of modified MB species, including MB without a sulfate group (Δ*mbnS*) and Cu-bound MB (Δ*mbnP* and Δ*mbnH*). Together, these results provide functional insight into the *mbn*-SB2 gene cluster and elucidate the genes involved—*mbnB*, *mbnC*, *mbnF*—in heterocyclic ring formation in MB-SB2.

**IMPORTANCE:**

MBs—particularly MB from *Methylocystis* sp. strain SB2—have great promise in treating human copper-related diseases, perhaps most notably Wilson disease. Understanding how the distinctive heterocyclic rings of MB that are responsible for copper binding are assembled is crucial to understanding MB biosynthesis, key information necessary to scale up production of MB-SB2 for medical applications. This study identifies the responsible genes for the post-translational modification (i.e., heterocyclic ring formation) of the MB-SB2 polypeptide precursor. In particular, these findings expand our mechanistic understanding of MB biosynthesis across different methanotrophs and highlight the diversity of enzymatic strategies used to generate copper-chelating heterocyclic rings.

## OBSERVATION

Methanobactins (MBs) are small, ribosomally synthesized, post-translationally modified polypeptides (RiPPs) produced by some aerobic methane-oxidizing bacteria (methanotrophs) for binding/collecting copper from the environment (1–5). MBs are distinguished by two heterocyclic rings in their structure that are critical for copper binding. Based partially on the type of heterocyclic rings, MBs can be divided into two groups. Group I MBs (e.g., MB of *Methylosinus trichosporium* OB3b, MB-OB3b) have two oxazolone rings as well as an internal disulfide bond (6), and Group II MBs (e.g., MB of *Methylocystis* sp. strain SB2, MB-SB2) have one oxazolone ring and one pyrazeindione or imidazolone ring, as well as a sulfonated threonine (7). The genes responsible (i.e., the *mbn* gene cluster) for MB biosynthesis, particularly the genes responsible for ring formation, have been extensively studied for *M. trichosporium* OB3b (8–12). However, knowledge about the *mbn* genes for any Group II MB has been severely limited, especially for MB-SB2 biosynthesis because of the lack of genetic tools for these methanotrophs. As MB-SB2 has significant therapeutic value in treating Wilson’s disease, a copper-related human disease (13), understanding the function of the genes in the *mbn*-SB2 gene cluster is important. Here, we report a systematic functional analysis of the *mbn*-SB2 gene cluster using a previously developed mutant strain of *M. trichosporium* OB3b that can heterologously express the *mbn*-SB2 gene cluster (14).

**Fig 1:**
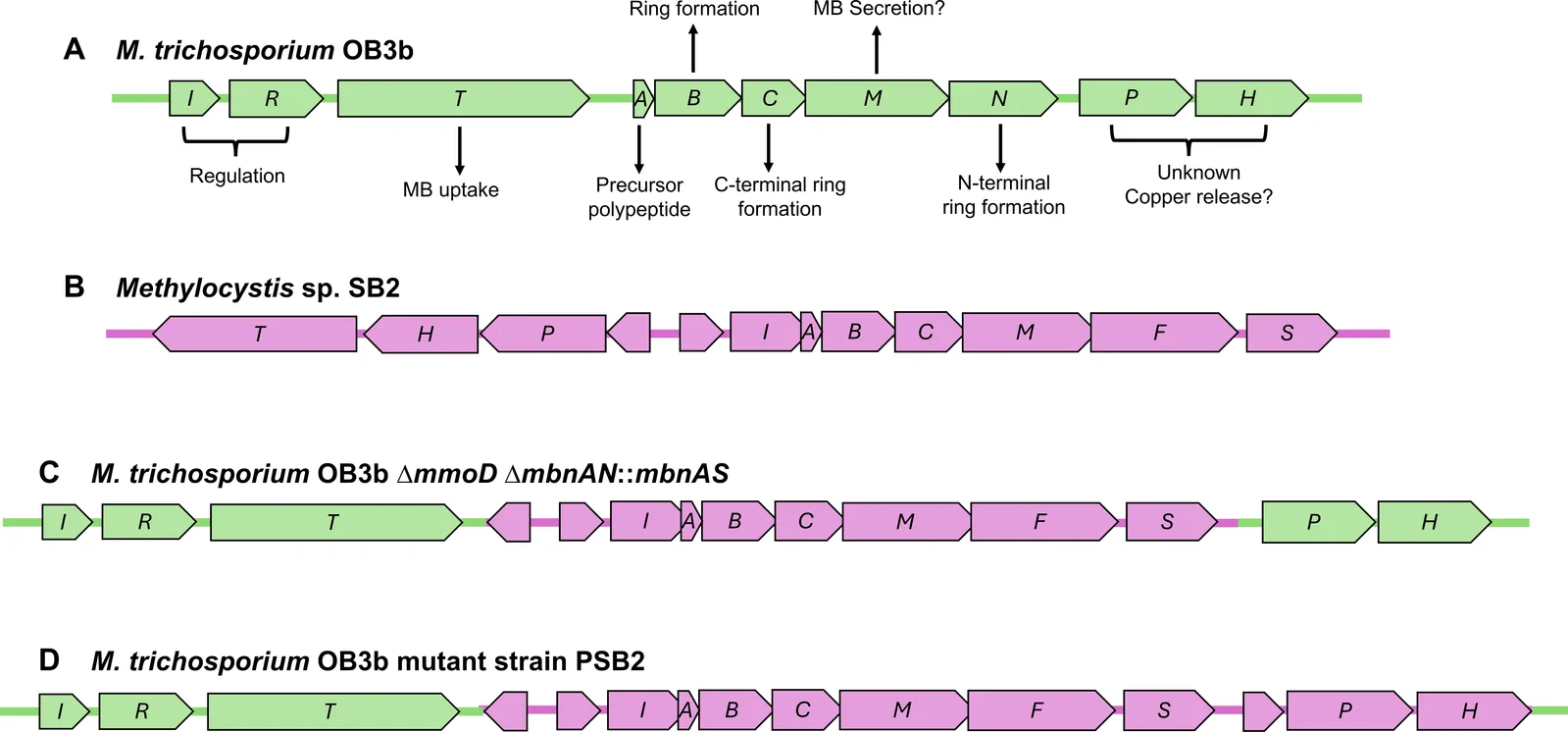
*mbn* gene cluster organization and mutant strain construction. Schematic of the *mbn* gene cluster of (**A**) *M. trichosporium* OB3b, (**B**) *Methylocystis* sp. SB2, (**C**) the previously described mutant strain of *M. trichosporium* OB3b ∆*mmoD* ∆*mbnAN::mbnAS* platform strain (14), and (**D**) strain PSB2 constructed in this study, which carries the complete *mbn*-SB2 gene cluster. Colored arrows indicate individual *mbn* genes; possible gene functions are labeled below each arrow.

To functionally investigate individual genes of the *mbn*-SB2 gene cluster, we first deleted the native *mbnP* and *mbnH* genes (*mbnPH*-OB3b) and inserted *mbnPH* of *Methylocystis* sp. strain SB2 into the genome of a previously described mutant strain of *M. trichosporium* OB3b (∆*mmoD* ∆*mbnAN::mbnAS*) (14), generating strain PSB2 that contains the complete *mbn*-SB2 gene cluster (Fig. 1). From strain PSB2, we systematically deleted each *mbn*-SB2 gene individually, generating mutants ∆*mbnB*, ∆*mbnC*, ∆*mbnM*, ∆*mbnF*, ∆*mbnS*, ∆*mbnP*, and ∆*mbnH*, and verified the genotype of each mutant by PCR and sequencing (Tables S1 and S2). For select mutants (∆*mbnB*, ∆*mbnC*, ∆*mbnF*), *mbn* genes were back-complemented and expressed using the broad-host-range vector pTJS-140 (15) under the control of the native *mbnA*-SB2 promoter to confirm the specific function of these genes (Fig. S1).

**Fig 2:**
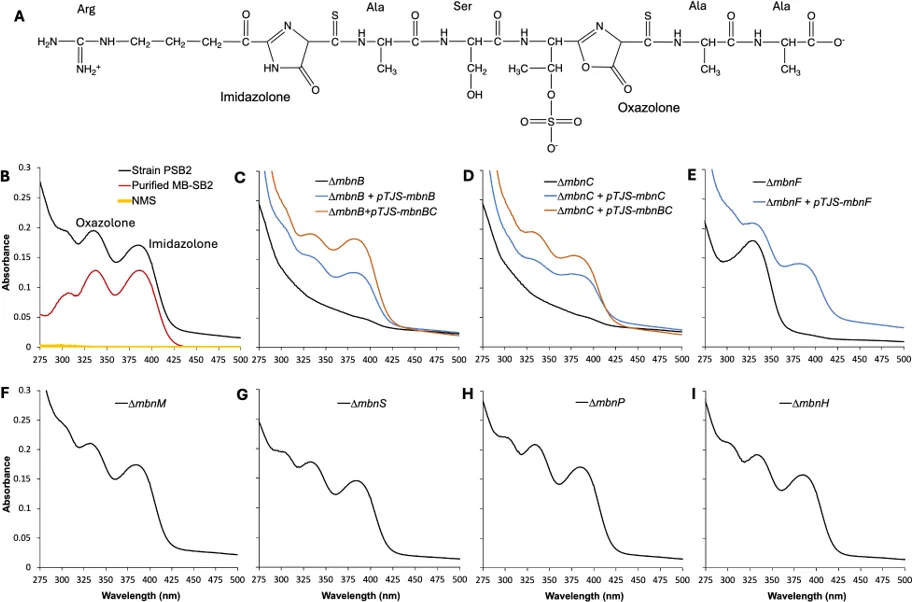
Chemical structure of MB-SB2 with the two heterocyclic rings indicated (**A**) and UV-vis absorption spectra of the supernatant of the *M. trichosporium* OB3b MB-SB2-producing mutant strain PSB2, purified methanobactin dissolved in sterile NMS medium (positive control) or sterile NMS medium as reference (negative control) (**B**), *mbnB* deletion and back-complementation mutants (**C**), *mbnC* deletion and back-complementation mutants (**D**), *mbnF* deletion and back-complementation mutants (**E**), *mbnM* deletion mutant (**F**), *mbnS* deletion mutant (**G**), *mbnP* deletion mutant (**H**), *mbnH* deletion mutant (**I**). The UV-vis absorption peaks for the oxazolone ring (336 nm) and imidazolone ring (386 nm) of MB-SB2 were indicated.

UV–vis spectrophotometry of culture supernatant was used to monitor MB-SB2 biosynthesis and ring formation (peak absorption of the oxazolone and imidazolone rings at 336, 386, and 386 nm) (Fig. 2A) (7).The supernatant of actively growing PSB2 contained MB-SB2 with both oxazolone and imidazolone rings as well as the expected molecular mass of MB-SB2 (~850.19, Table 1; Fig. S2), confirming the expression of the *mbn*-SB2 gene cluster and MB-SB2 biosynthesis (Fig. 2B). Deletion of either *mbnB* or *mbnC* disrupted MB-SB2 production, and neither ring was observed (Fig. 2C and D). The liquid chromatography–mass spectrometry (LC–MS) analysis showed formation of an unidentified MB with a molecular mass of ~1,125.34 (Table 1; Fig. S2). Back-complementation of ∆*mbnB* or ∆*mbnC* with the respective gene (*mbnB* and *mbnC*) on pTJS-140 vector restored MB-SB2 biosynthesis, though absorption peaks (especially the oxazolone ring) were weaker than that in PSB2 (Fig. 2C and D). Such weaker MB-SB2 production may be due to the separation of *mbnB* and *mbnC* genes that interrupted their original co-transcription and formation of a MbnB–MbnC complex (Fig. S3). Indeed, co-expression of *mbnBC* in ∆*mbnB* and ∆*mbnC* improved MB-SB2 production (Fig. 2C and D), consistent with the known protein and protein interaction of MbnB and MbnC for the C-terminal oxazolone ring formation (10).

**TABLE 1:** Proposed structures of methanobactin forms produced by various MB mutants and their masses (ND, not determined)

Strain	Proposed structure(s)	Predicted mass	Measured mass	Mass error (ppm)
PSB2	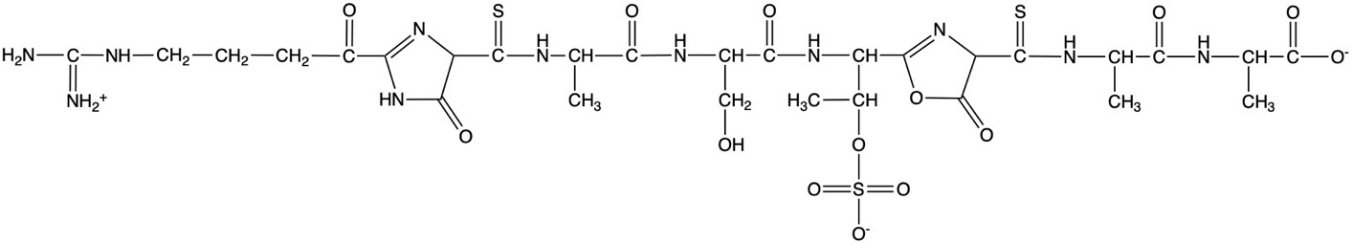	850.1924	850.1932	0.9
Δ*mbnB*	Unknown	ND	1,125.3484	ND
Δ*mbnC*	Unknown	ND	1,125.3489	ND
Δ*mbnF*	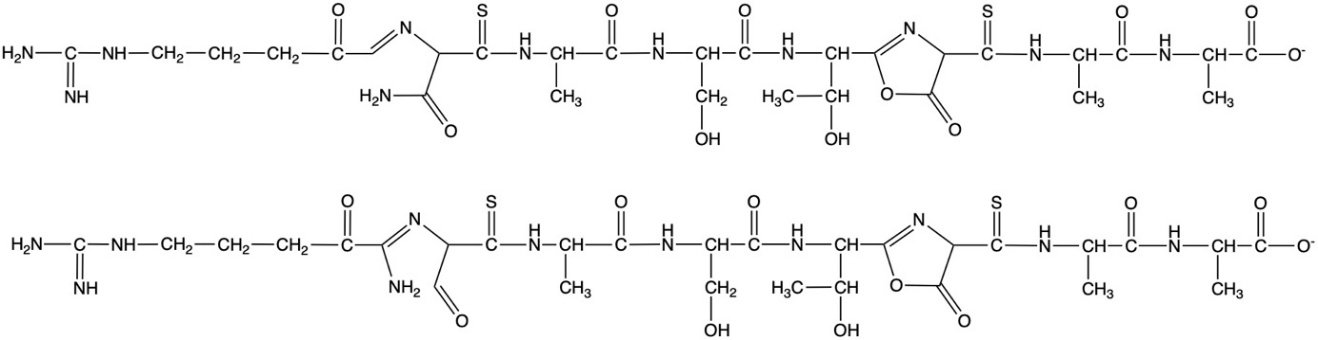	772.2512	772.2513	0.1
Δ*mbnM*	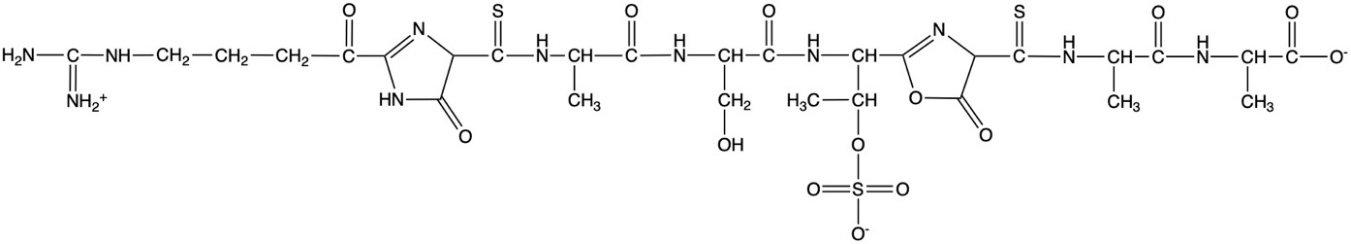	850.1924	850.1929	0.6
	+ Unknown	ND	824.2145	ND
Δ*mbnS*	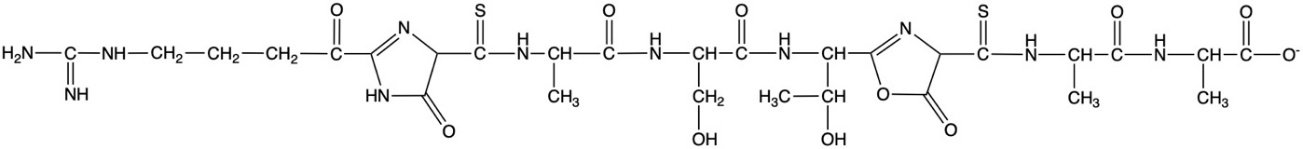	770.2356	770.2358	0.26
Δ*mbnP*	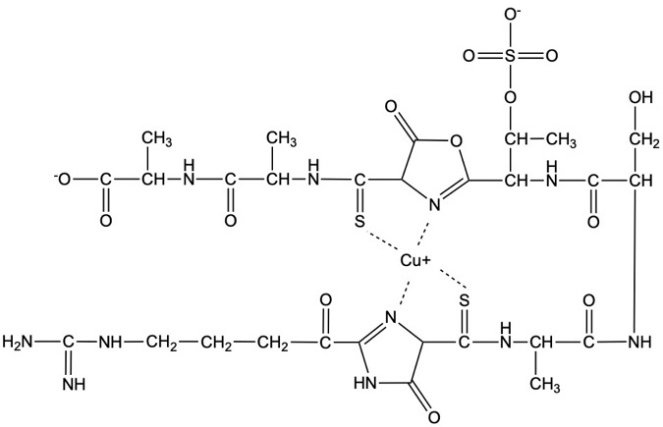	912.1142	912.1096	−5.0
	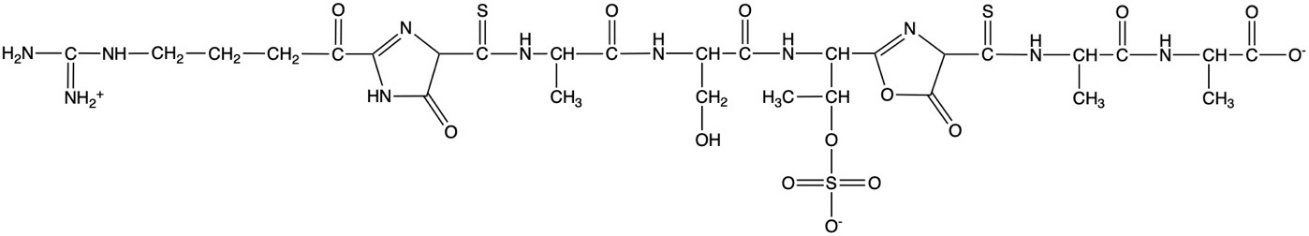	850.1924	850.1956	3.8
Δ*mbnH*	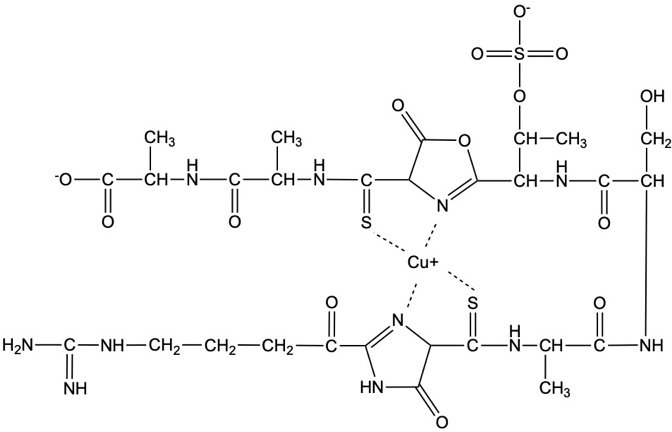	912.1142	912.1093	−5.4
	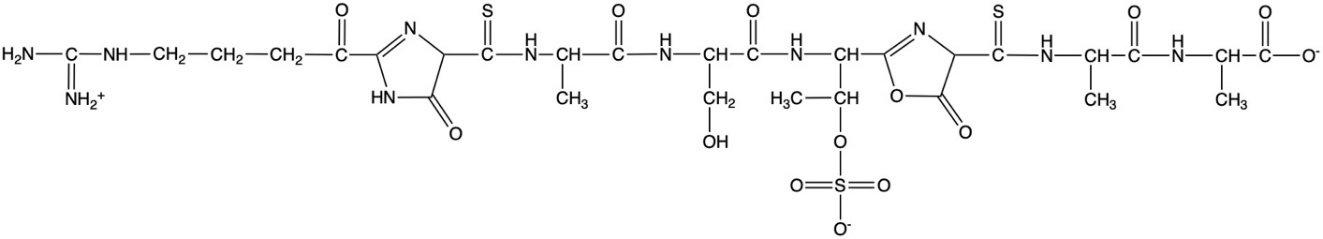	850.1924	850.1954	3.5

A qualitatively different phenotype was observed for the ∆*mbnF* mutant. *mbnF* encodes for an NADPH-dependent flavin monooxygenase that is absent from the *mbn*-OB3b gene cluster (16). Interestingly, deletion of the *mbnF* in PSB2 resulted in formation of an MB that lacked the imidazolone ring, while evidence of the oxazolone ring was observed (Fig. 2E). This MB exhibited a molecular mass of 772.25, indicating a partially formed imidazolone ring and the absence of the sulfate group (Table 1; Fig. S2). Back-complementation of ∆*mbnF* with *mbnF*-pTJS-140 restored formation of MB-SB2 with both heterocyclic rings (Fig. 2E). The result indicates that *mbnF* is specifically required for imidazolone ring formation in MB-SB2. Moreover, together with the result from ∆*mbnB* and ∆*mbnC*, these data show that MbnF specifically acts on MB bearing the C-terminal oxazolone ring (mediated by MbnBC), rather than an unmodified MB precursor as neither ∆*mbnB* nor ∆*mbnC* can make a MB with only the N-terminal imidazolone ring. This substrate ordering for biosynthesis of MB-SB2 is different from MB-OB3b (9), suggesting the responsible enzymes (MbnF and MbnN-OB3b) for the N-terminal rings formation in MB-SB2 and MB-OB3b are different in terms of their substrate specificity. MbnN of the OB3b-MB gene cluster encoding for an aminotransferase may act on either the precursor or the C-terminal oxazolone ring-modified intermediate of MB-OB3b (8, 9). Previous studies indicate aminotransferases commonly display broad and multi-substrate specificity (17, 18),

In contrast, deletion of *mbnM*, *mbnS*, *mbnP*, or *mbnH* in PSB2 had no effect on MB-SB2 ring formation (Fig. 2F through I), indicating that none of these genes is required for such post-translational modifications. Among these, the MB produced by the ∆*mbnS* mutant showed a molecular mass of 770.23, consistent with MB-SB2 lacking the sulfate group (Table 1; Fig. S2). This result further confirmed MbnS as a sulfotransferase that is involved in the sulfunotion of MB. In contrast, the MB produced by the ∆*mbnM*, ∆*mbnP*, and ∆*mbnH* mutants all displayed the expected molecular mass of MB-SB2 (~850.19; Table 1; Fig. S2). It should be stressed, however, that the supernatant of the ∆*mbnM* mutant also had another compound of unknown structure with mass of 824.21. Furthermore, the supernatant of the ∆*mbnP* and ∆*mbnH* mutants also contained a significant amount of copper-bound MB-SB2, with molecular mass of 912.11 (Table 1; Fig. S2). Such high amount of copper-bound MB was not observed in the production strain PSB2, suggesting a function of MbnP and MbnH (MbnPH) in copper release from MB and/or in uptake of copper-containing MB-SB2. Moreover, it is particularly interesting that MB was observed in the spent medium of ∆*mbnM*, as this gene is predicted to encode for a MATE protein that was speculated to be involved in MB secretion (1, 2). That MB-SB2 was observed in this mutant suggests that *mbnM* is either not involved in MB secretion or that there are multiple means by which MB is released from the cell. Finally, it should be stressed that RT-PCR confirmed that all *mbn*-SB2 genes were expressed in PSB2 and in the respective mutants (except the deleted genes themselves). The *mbn* genes carried by pTJS-140 vector were also expressed (Fig. S4).

In summary, this study provides the first systematic genetic dissection of the *mbn*-SB2 gene cluster and establishes the following: (i) *mbnBC* is required for C-terminal oxazolone ring formation in MB-SB2, consistent with its conserved role for MB biosynthesis; (ii) *mbnF* is specifically responsible for N-terminal imidazolone ring formation and requires the C-terminal oxazolone ring intermediate as its substrate; and (iii) *mbnM*, *mbnS*, *mbnP*, and *mbnH* are not involved in ring formation but are involved in other modifications of MB, including sulfunotion (*mbnS*) and copper release (*mbnPH*). These findings deepen our understanding of the diversity of enzymatic strategies used in MB biosynthesis across methanotrophs and provide guidance for genetic engineering of methanotrophs for MB-SB2 production for medical applications.

## SUPPLEMENTAL MATERIAL

File S1 spectrum.01622-26-s0001.xlsx Mass spectra data.

Supplemental materials spectrum.01622-26-s0002.pdf Supplemental methods, Tables S1 and S2, and Figs. S1 to S4.

ASM does not own the copyrights to Supplemental Material that may be linked to, or accessed through, an article. The authors have granted ASM a non-exclusive, world-wide license to publish the Supplemental Material files. Please contact the corresponding author directly for reuse.
