# Supplementary material for: Functional analysis of the methanobactin-encoding gene cluster of Methylocystis sp. strain SB2
Source: Microbiol Spectr. 2026 Sep 18;14(11):e01622-26. doi: 10.1128/spectrum.01622-26 (PMC13729199; doi:10.1128/spectrum.01622-26)
Supplement: Supplementary file 1 [file spectrum.01622-26-s0002.pdf]

Supplementary Information

Materials and Methods

Methanotrophs strains and growth condition

The methanotrophs strains/mutants used in this study were constructed from a previously described *M. trichosporium* OB3b Δ*mmoD* Δ*mbnAN*::*mbnAS*, where part of the *mbn* gene cluster of *Methylocystis* sp. SB2 was inserted into the chromosome of *M. trichosporium* OB3b (1). A mutant strain with the complete genes of *mbn*-SB2 gene cluster in its genome (strain PSB2) (Fig 1) was first constructed and then various mutants with each *mbn*-SB2 gene deleted were constructed from strain PSB2 (Table S1). Wild type *Methylosinus trichosporium* OB3b and the constructed mutants were grown in nitrate mineral salt (NMS) medium (2) with or without 25 µg/ml kanamycin and in the absence (no added) or presence of copper (1 µM as $CuCl_2$). Methane and air were added at a methane-to-air ratio of 1:2. Cultures were incubated in the dark at 30°C. When methanol was used as the carbon source, HPLC plus grade (> 99%) methanol was added to NMS medium at a concentration of 0.25% (v/v). Liquid cultures were grown in 250-ml sidearm Erlenmeyer flasks with 20 ml NMS medium with shaking at 200 rpm. Growth was monitored non-invasively by measuring the optical density at 600 nm (OD_{600}) with a Genesys 20 visible spectrophotometer via the side-arm (Spectronic Unicam, Waltham, MA). Triplicate biological cultures were prepared for all experimental conditions. Cultures were harvested at middle to late exponential phase (OD_{600} of ~0.75) for RNA isolation and transcriptional analysis of specific *mbn*-SB2 gene expression. *Escherichia coli* was grown in Luria-Bertani broth (LB) at 37°C with or without supplement of 25 µg/ml kanamycin.

Knock-in of *mbnPH* of *Methylocystis* sp. SB2 into the genome of a previously constructed MB-SB2-producing platform *M. trichosporium* OB3b Δ*mmoD* Δ*mbnAN::mbnAS*

M. trichosporium OB3b Δ*mmoD* Δ*mbnAN*::*mbnAS* (Fig 1) was constructed in an earlier study for heterologous expression and production of MB-SB2 in *M. trichosporium* OB3b (1). To investigate the function of genes in the *mbn*-SB2 gene cluster, we first inserted the *mbnPH* genes of *Methylocystis* sp. SB2 into the genome of Δ*mmoD* Δ*mbnAN*::*mbnAS* (Fig 1). That is, *mbnPH*-SB2 were inserted with replacement of the native *mbnPH* genes of Δ*mmoD* Δ*mbnAN*::*mbnAS* using a sucrose counter-selection protocol (3). Briefly, *mbnPH*-SB2 and the upstream and downstream regions (arms) of *mbnPH* of *M. trichosporium* OB3b were PCR amplified using the primers listed in Table S2. The arms and *mbnPH* were ligated together and then inserted into the pK18mobsacB mobilizable suicide vector (pre-digested with HindIII restriction enzyme) (4) using NEBuilder® HiFi DNA Assembly Cloning Kit. pK18mobsacB vector with *mbnPH* of *Methylocystis* sp. strain SB2 and associated arms was transferred to *E. coli* Top10 for production (Invitrogen, Carlsbad, CA). The plasmid was then extracted from transformed *E. coli* Top-10 using the Plasmid Mini Kit (Qiagen, Hilden, Germany) following the manufacturer's instructions, and then sent to Eurofins for Whole Plasmid Sequencing to confirm the accuracy of the PCR and cloning. After sequence confirmation, the plasmid was then transformed to *E. coli* S17-1 (5) for conjugation. Conjugation of *E. coli* S17-1 carrying the constructed vector with *M. trichosporium* OB3b Δ*mmoD* Δ*mbnAN::mbnAS* was performed as described by Martin and Murrell (6). Transconjugants were grown on NMS plates supplemented with 25 µg/ml kanamycin and 10 µg/ml nalidixic acid. The generated kanamycin resistant transconjugant

colonies (after 10 days of incubation) were transferred to NMS plates with kanamycin and incubated for 7 days, and subsequently transferred to NMS plates with 2.5% sucrose (mass/vol). Sucrose-resistant colonies were generated after 10 days of incubation and were screened for knock-in of *mbnPH* by colony PCR. Successful *mbnPH* knock-in mutants (strain PSB2) where the entire *mbn* gene cluster from *Methylocystis* sp. strain SB2 (*mbnABCFMFS* + *mbnPH* + *mbnI* + unknown12) were inserted in the chromosome of *M. trichosporium* OB3b (while also removing native *mbnPH*) were further confirmed by PCR with DNA extracted from the mutant using the DNeasy PowerSoil Pro Kit (Qiagen, Hilden, Germany) following manufacturer's instructions.

The construction of other *M. trichosporium* OB3b mutants with specific genes deleted/modified (Table S2) was then performed following a similar procedure as described above, except the vector was re-constructed with appropriate arms and conjugation accepter was replaced with strain PSB2. The desired *mbnA* sequences (for mutation of the *mbnA* in strain PSB2) were synthesized at Integrated DNA Technologies (IDT).

For back complementation of genes deleted in strain PSB2 (i.e., Δ*mbnB*, Δ*mbnC*, Δ*mbnF*), *mbnB*/*C*/*F* gene from *Methylocystis* sp. SB2 was PCR amplified using the primers listed in Table S1. The promoter of *mbnI/mbnA* (Fig 1) was used for control the transcription of *mbnB/C/F*. *mbnB*/*C*/*F* together with the promoter was cloned into pTJS-140 broad-host-range cloning vector (pre-digested with KpnI restriction enzyme) (Fig S1) (7) using NEBuilder® HiFi DNA Assembly Cloning Kit. The pTJS-140 vector with *mbnB*/*C*/*F* was transformed to *E. coli* S17-1 as described above. *E. coli* S17-1 with the constructed plasmid was conjugated with strain PSB2. Transconjugants were grown on NMS plates supplemented with 25 µg/ml kanamycin and 10 µg/ml nalidixic acid. Selection and conformation of Δ*mbn B*/*C*/*F* with pTJS- *mbnB*/*C*/*F* were done as described above.

UV-vis analysis of MB-SB2 production in the mutants

Mutants were grown with methane or methanol in NMS medium without copper. The supernatant of the cultures was collected when OD_{600} was ~ 1.5. The UV-Vis absorption spectra (275 – 500 nm) of the supernatant were measured on a Specord Plus UV-Vis Spectrophotometer (analytik-jena, Germany).

RNA isolation and reverse transcription PCR (RT-PCR)

RNA isolation was performed with a bead-beating procedure followed by column purification using an RNeasy Mini Kit (Qiagen, Hilden, Germany) as described before (8). Genomic DNA was removed from the column with RNase-free DNase (Qiagen, Hilden, Germany) treatment. Absence of genomic DNA was confirmed by 16S rRNA gene targeted PCR with extracted RNA samples as templates. Purified RNA was quantified using a NanoDrop 1000 Spectrophotometer (Thermo Scientific, Wilmington, DE). cDNA was synthesized from 200 ng total RNA using SuperScript™ III Reverse Transcriptase (Invitrogen, Carlsbad, CA) following manufacturer's instructions.

RT-PCR was performed to verify expression of *mbn* genes of *Methylocystis* sp. SB2 knocked-in various mutants (Table 1). Primers for RT-PCR were listed in Table S1. RT-PCR was performed

with 1 µl cDNA and the following program: 95°C for 5 min, followed by 30 cycles of 95°C for 30 s, 55°C for 30 s and 72°C for 30 s, and final extension at 72°C for 5 min.

Liquid chromatography-mass spectroscopy (LC-MS)
Mutants were grown with methanol in NMS medium without copper as described above. The supernatant of the cultures was collected when OD_{600} was ~ 1.5. Separation of MB from the supernatant was achieved using a UltiMate 3000 UHPLC system (Thermo Scientific) with a Hypersil GOLD™ C18 Selectivity HPLC Column (3 µm, 50 mm × 2.1 mm). The gradient separation was performed with a 5-100% mobile phase (0.1% formic acid/methanol) gradient and a stationary phase of 0.1% formic acid in water.

Mass was determined in the negative ion mode on a Q Exactive Orbitrap Mass Spectrometer (Thermo Scientific) coupled to the UltiMate 3000 UHPLC system. The mass spectrometer was operated in ESI negative resolution mode in the range 650-1200 Da. The capillary voltage was set to 2.5 kV with a sampling cone voltage of 40V, a source temperature of 100 ºC and a desolvation temperature of 235 ºC. The cone gas was set to 50 L/h with desolvation gas at 600 L/h and the nebulizer set to 6.5 bar.

Table S1. *M. trichosporium* OB3b mutant strains used in this study.

Strain	Description	Reference
Δ*mmoD* Δ*mbnAN::mbnAS*	Δ*mmoD* with *mbnAS* of *Methylocystis* sp. SB2 knocked in its genome and replaced its original *mbnAN* (Fig 1)	(1)
Δ*mmoD* Δ*mbnAN::mbnASPH* (strain PSB2)	Δ*mmoD* Δ*mbnAN::mbnAS* with *mbnPH* of *Methylocystis* sp. SB2 knocked in its genome and deletion of native *mbnPH* of *M. trichosporium* OB3b (Fig 1)	This study
Δ*mbnB*	Strain PSB2 with *mbnB* deleted	This study
Δ*mbnB* + pTJS-*mbnB*	Δ*mbnB* carrying pTJS-140 with *mbnB* of *Methylocystis* sp. SB2	This study
Δ*mbnB* + pTJS-*mbnBC*	Δ*mbnB* carrying pTJS-140 with *mbnBC* of *Methylocystis* sp. SB2	This study
Δ*mbnC*	Strain PSB2 with *mbnC* deleted	This study
Δ*mbnC* + pTJS-*mbnC*	Δ*mbnC* carrying pTJS-140 with *mbnC* of *Methylocystis* sp. SB2	This study
Δ*mbnC* + pTJS-*mbnBC*	Δ*mbnB* carrying pTJS-140 with *mbnBC* of *Methylocystis* sp. SB2	This study
Δ*mbnM*	Strain PSB2 with *mbnM* deleted	This study
Δ*mbnF*	Strain PSB2 with *mbnF* deleted	This study
Δ*mbnF* + pTJS-*mbnF*	Δ*mbnF* carrying pTJS-140 with *mbnF* of *Methylocystis* sp. SB2	This study
Δ*mbnS*	Strain PSB2 with *mbnS* deleted	This study
Δ*mbnP*	Strain PSB2 with *mbnP* deleted	This study
Δ*mbnH*	Strain PSB2 with *mbnH* deleted	This study

Table S2. Primers used in this study.

Primer name	Sequence (5'—3') [a]	Application	Reference
mbnB-armA_F<br>mbnB-armA_R	agtcgacctgcaggcatgcaGCGCGGCTATGTGTCGAAG<br>tcagatcgacCTGAGCCATGTCATAGGTGTC	PCR	This study
mbnB-armB_F<br>mbnB-armB_R	catggctcagGTCGATCTGAAGGCGTTGC<br>taaaacgacggccagtgccaGGTGACCGCGACATCGAG	PCR	This study
mbnC-armA_F<br>mbnC-armA_R	agtcgacctgcaggcatgcaAGGCTGATCGCCGAAGGG<br>gaaatatgtcCATCAGCGCAGGGTAAAGG	PCR	This study
mbnC-armB_F<br>mbnC-armB_R	tgcgctgatgGACATATTTCCGGGGTGGCAGAGC<br>taaaacgacggccagtgccaGCGTTGGCCGTTCGCGGC	PCR	This study
mbnM-armA_F<br>mbnM-armA_R	agtcgacctgcaggcatgcaGACACGCTCGCCTTCCTC<br>acgggacgccGATGAAGGCGGCTGCGGA	PCR	This study
mbnM-armB_F<br>mbnM-armB_R	cgccttcatcGGCGTCCCGTGGCCCGCT<br>taaaacgacggccagtgccaCGTTCTTCGTCGAAATCGGACGCCTGATC<br>G	PCR	This study
mbnF-armA_F<br>mbnF-armA_R	agtcgacctgcaggcatgcaTCGCCTCGTCTTTTTGGTTATGG<br>cgcgcgcaaaAAGCAACGTCGCCGCAGA	PCR	This study
mbnF-armB_F<br>mbnF-armB_R	gacgttgctt TTTGCGCGCGTGCGCGGT<br>taaaacgacggccagtgccaTCTCTGCCGAATTTCTCGAACAGCAGGC	PCR	This study
mbnS-armA_F<br>mbnS-armA_R	agtcgacctgcaggcatgcaTCCACGCGCGATCAGGCG<br>atgtcgcctcTCGACCACTCTCCTTTGCGAAGAC	PCR	This study
mbnS-armB_F<br>mbnS-armB_R	gagtggtcgaGAGGCGACATTCCTCTAGAATCC<br>taaaacgacggccaGTGCCAGTCCCCCGTCGTGAGAGG	PCR	This study
mbnP-armA_F<br>mbnP-armA_R	agtcgacctgcaggcatgcaCTAAAGACTCTGCGCGCTCAG<br>cgaacatcggACCGGCGATAACGGCGGT	PCR	This study
mbnP-armB_F<br>mbnP-armB_R	tatcgccggtCCGATGTTCGAAAATCTCGGGC<br>taaaacgacggccagtgccaAGTGGTGGCACTCGGCCT	PCR	This study
mbnH-armA_F<br>mbnH-armA_R	agtcgacctgcaggcatgcaGCGCCGACATCAACGACACG<br>acgactcgtcCCAGGACTCCGCGCTGGG	PCR	This study
mbnH-armB_F<br>mbnH-armB_R	ggagtcctggGACGAGTCGTTGCTGACATC<br>taaaacgacggccagtgccaTGATCCGAGCCGACGACAG	PCR	This study
Promoter-F [b]<br>Promoter-R	AGTGAAGCAGGCTCGCCG<br>GGATTGCCGCCAACCATTG	PCR	This study
mbnB-F [b]<br>mbnB-R	gcggcaatccGGACGGTTTGCTTGCGCC<br>tctagaggatccccgggtacCTAGGCAGTGAGAGCAAGGTC	PCR	This study
mbnC-F [b]<br>mbnC-R	gcggcaatccTCAAATCGAGTTCGACGATATCGTC<br>tctagaggatccccgggtacTCATCGCGCGAGGCTCTG	PCR	This study
mbnF-F [b]<br>mbnF-R	gcggcaatccATGAGCGTGGAGCGAGTC<br>tctagaggatccccgggtacCTAGCTTGCGTCGGCGAAC	PCR	This study
mbnA_F<br>mbnA_R	AGCGCATCACCCTCAATGTC<br>ATGTCATAGGTGTCGCCGAG	RT-PCR	(1)
mbnB_F<br>mbnB_R	CCAGTCGGGTTGCACATTAT<br>CCATGATGGACGGGTAATTCTC	RT-PCR	(1)

mbnC_F<br>mbnC_R	CGGACTGATCGCGACCTTTA<br>CGGAAAATCGACATGCCGTC	RT-PCR	(1)
mbnM_F<br>mbnM_R	CGCAGAAATTCCAGCGAAAG<br>CAGCCGACTCCATAACCAAA	RT-PCR	(1)
mbnF_F<br>mbnF_R	GAGTCCCGGTTCTGATCGTC<br>CAAACAACGCGTCCTCCAAG	RT-PCR	(1)
mbnS_F<br>mbnS_R	AAGCGTCAATTCCGCTCCTA<br>GACGTGCGGATGCTGAAAAA	RT-PCR	(1)
mbnP_F<br>mbnP_R	CGCGCATTCACTCGATTTAC<br>CCAGTCGTTCTGTTCCAGTT	RT-PCR	(1)
mbnH_F<br>mbnH_R	GGACTTTGCCCGACTTCAT<br>CCATCTGCCTTTCCAGTGTTA	RT-PCR	(1)

[a] Lowercase letters indicate overlap sequence with vectors (pK18mobsacB or pTJS-140) included in these primers.

[b] Primers used for constucting the pTJS-140 vector for expressing the *mbnB*, *mbnC*, *mbnF* genes

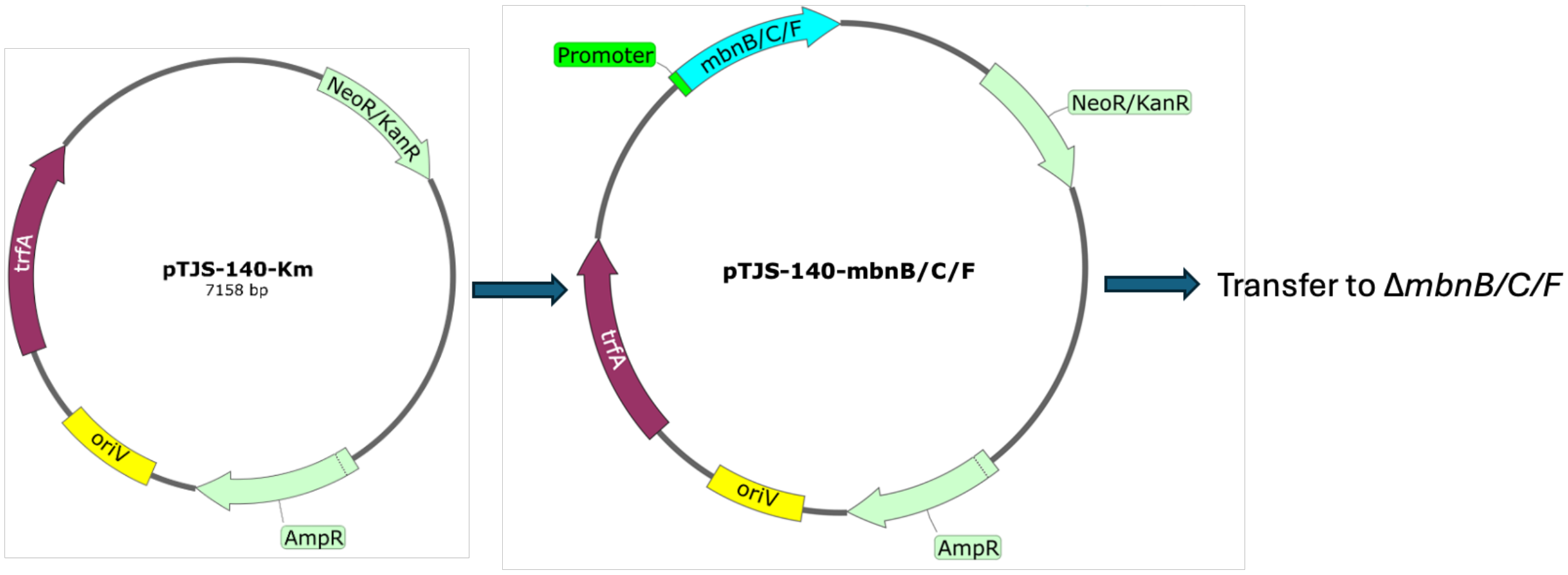

Fig S1. Construction of the pTJS-140 vector for expressing *mbn*B/C/F in Δ*mbnB/C/F*.

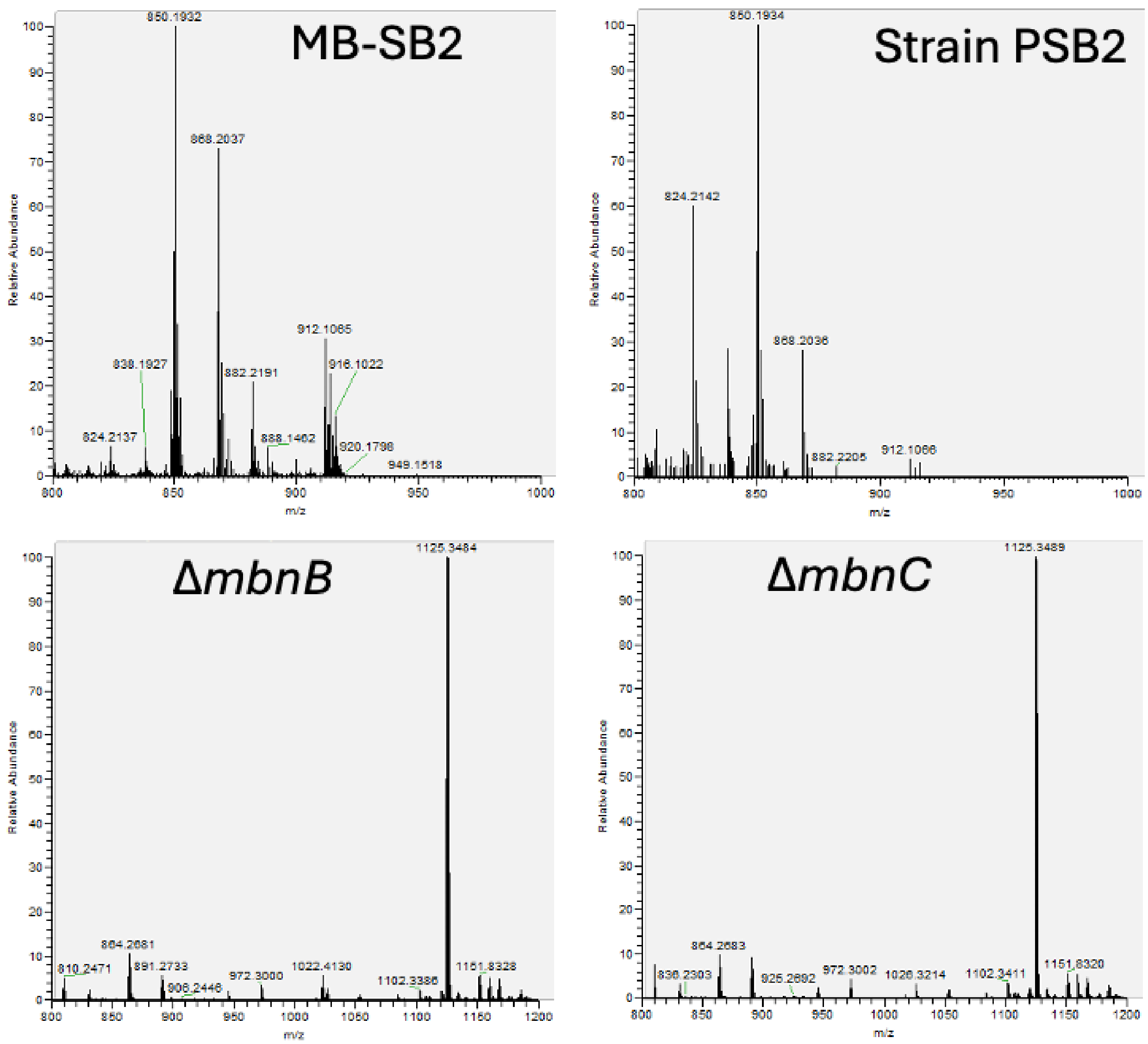

Fig S2. Mass spectrum in negative ion mode of purified MB-SB2 and MB separated from the supernatant of specific *mbn* deletion mutants.

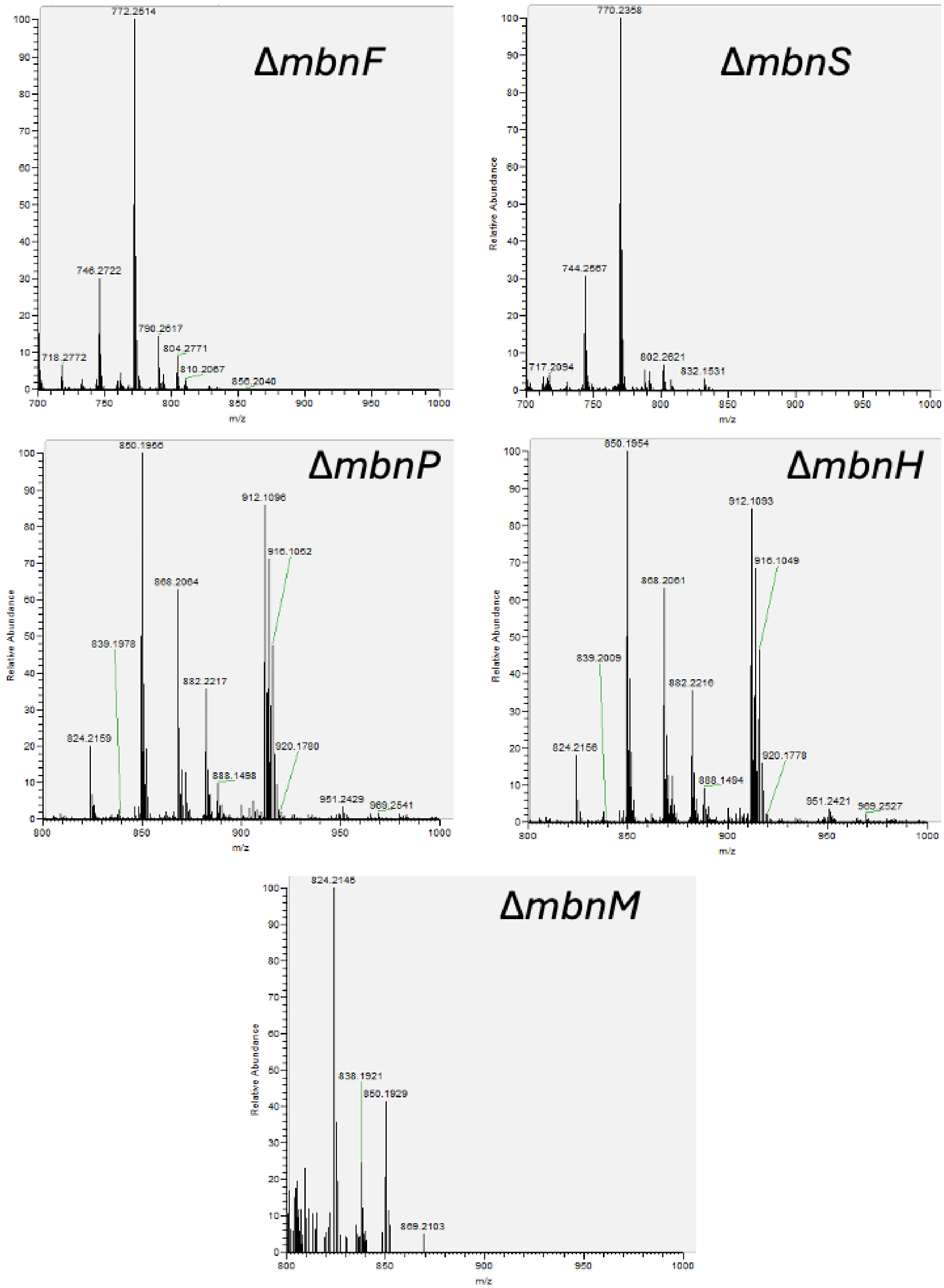

Fig S2 Continued

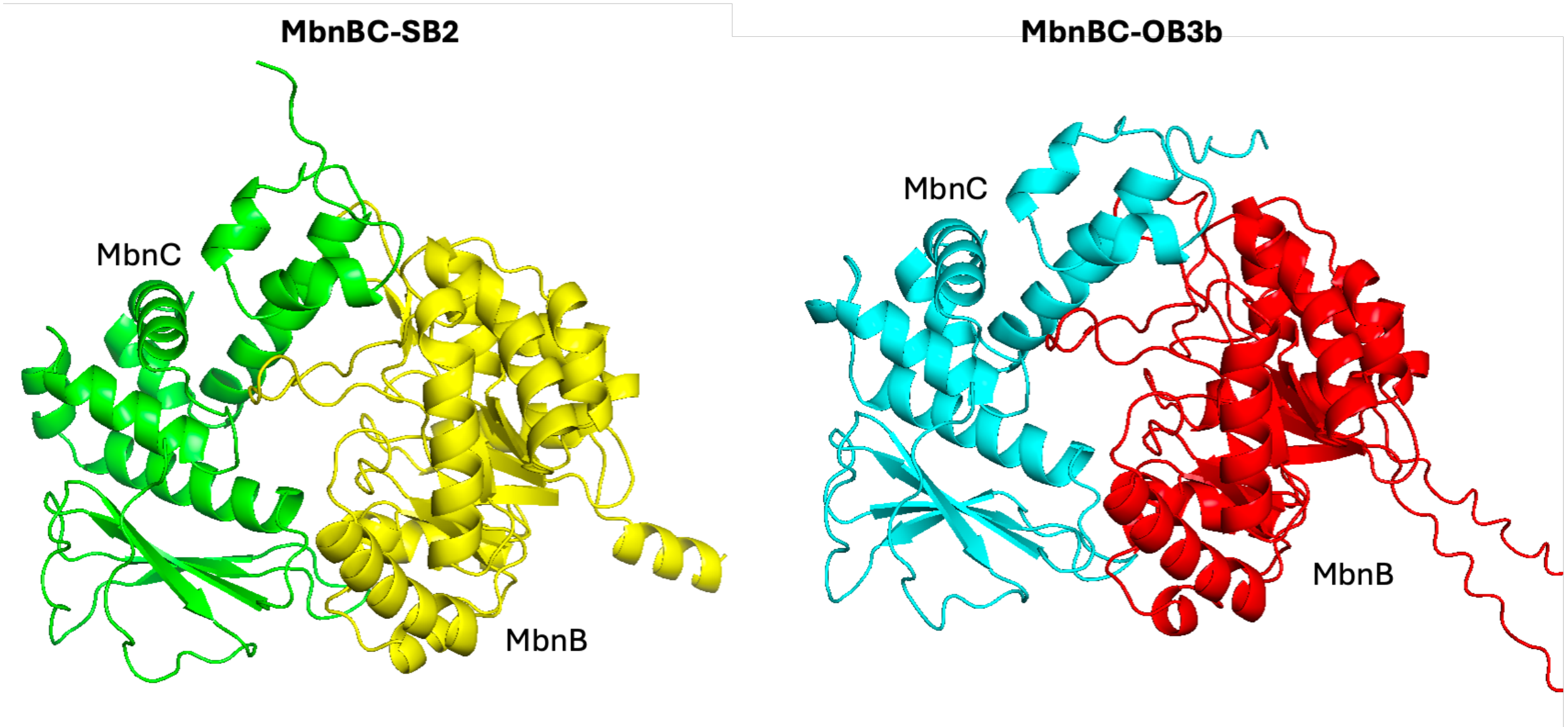

Fig S3. Comparison of the structure of the MbnBC complex of *Methylocystis* sp. SB2 (predicted by Alphafold-3) and *M. trichosporium* OB3b.

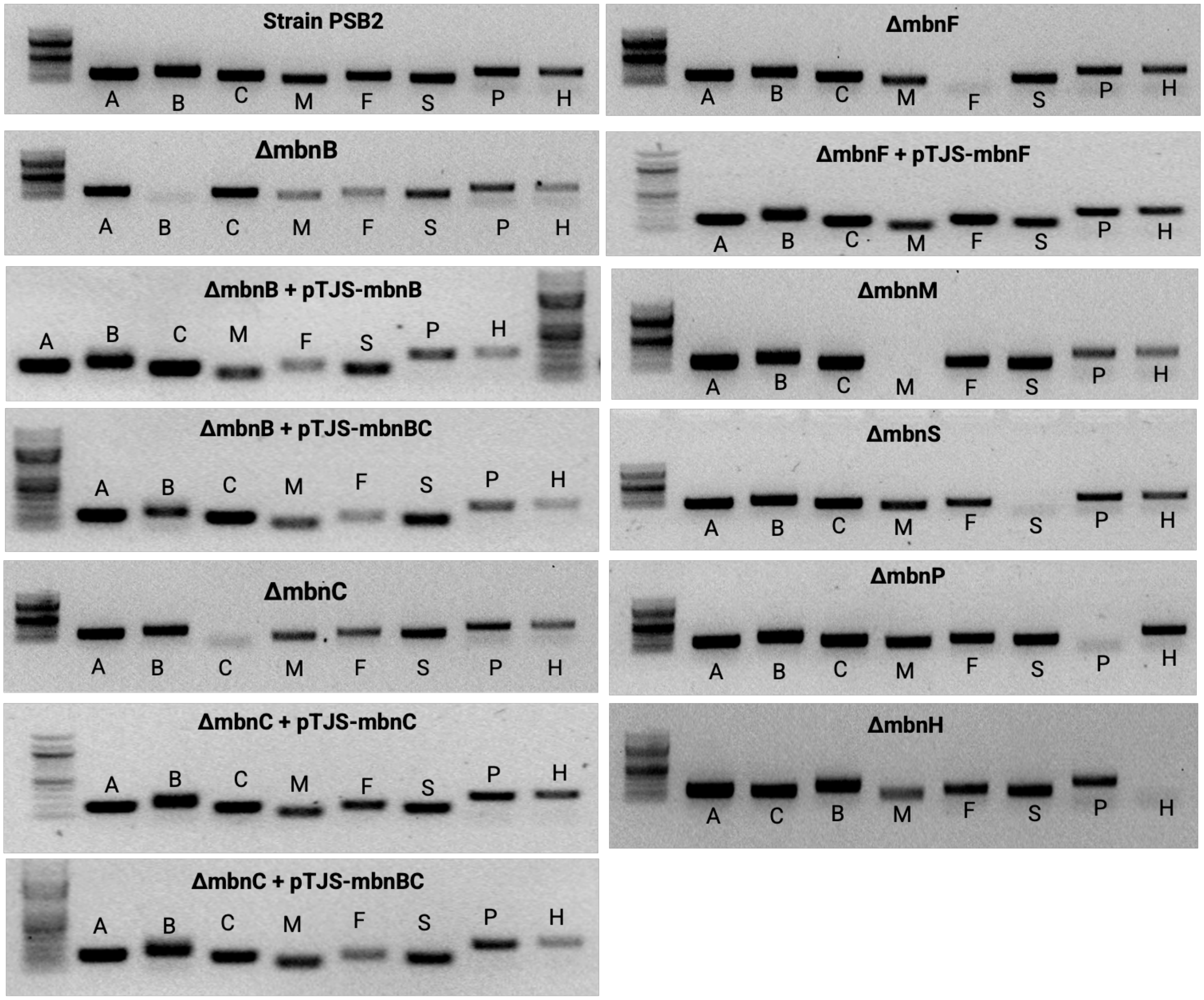

Fig S4. RT-PCR analysis of the *mbn* genes expression in strain PSB2 and its mutants with specific *mbn* genes deleted and back complemented.

References

1. Peng P, DiSpirito AA, Lewis BJ, Nott JD, Semrau JD. 2024. Heterologous Biosynthesis of Methanobactin from Methylocystis sp. Strain SB2 in Methylosinus trichosporium OB3b. ACS Synthetic Biology 13:2347-2356.
2. Whittenbury R, Phillips K, Wilkinson J. 1970. Enrichment, isolation and some properties of methane-utilizing bacteria. Microbiology 61:205-218.
3. Welander PV, Summons RE. 2012. Discovery, taxonomic distribution, and phenotypic characterization of a gene required for 3-methylhopanoid production. Proc Natl Acad Sci U S A 109:12905-12910.
4. Schäfer A, Tauch A, Jäger W, Kalinowski J, Thierbach G, Pühler A. 1994. Small mobilizable multi-purpose cloning vectors derived from the *Escherichia coli* plasmids pK18 and pK19: selection of defined deletions in the chromosome of *Corynebacterium glutamicum*. Gene 145:69-73.
5. Simon R. 1984. High frequency mobilization of gram-negative bacterial replicons by the in vitro constructed Tn 5-Mob transposon. Mol Gen Genet 196:413-420.
6. Martin H, Murrell J. 1995. Methane monooxygenase mutants of *Methylosinus trichosporium* constructed by marker-exchange mutagenesis. FEMS Microbiol Lett 127:243-248.
7. Smith TJ, Slade SE, Burton NP, Murrell JC, Dalton H. 2002. Improved system for protein engineering of the hydroxylase component of soluble methane monooxygenase. Appl Environ Microbiol 68:5265-5273.
8. Peng P, Zheng Y, Koehorst JJ, Schaap PJ, Stams AJ, Smidt H, Atashgahi S. 2017. Concurrent haloalkanoate degradation and chlorate reduction by *Pseudomonas chloritidismutans* AW-1^{T}. Appl Environ Microbiol 83:e00325-17.
